# The Additive Effect of CBT Elements on the Video Game ‘Mindlight’ in Decreasing Anxiety Symptoms of Children with Autism Spectrum Disorder

**DOI:** 10.1007/s10803-021-04927-8

**Published:** 2021-03-03

**Authors:** Lieke A. M. W. Wijnhoven, Rutger C. M. E. Engels, Patrick Onghena, Roy Otten, Daan H. M. Creemers

**Affiliations:** 1 Present Address: Mental Health Care Institute Karakter, Reinier Postlaan 12, 6525 GC Nijmegen, The Netherlands; 2 Erasmus School of Social and Behavioral Sciences, Erasmus University, P.O. Box 1738, 3000 DR Rotterdam, The Netherlands; 3 Katholieke Universiteit Leuven, P.O. Box 5005, 3000 Leuven, Belgium; 4 Present Address: Behavioural Science Institute, Radboud University, P.O. Box 9104, 6500 HE Nijmegen, The Netherlands; 5 Present Address: REACH Institute, Arizona State University, P.O. Box 876005, Tempe, AZ USA; 6 Mental Health Care Institute GGZ Oost-Brabant, P.O. Box 3, 5427 ZG Boekel, The Netherlands; 7 Mental Health Care Institute Pluryn, P.O. Box 53, 6500 AB Nijmegen, The Netherlands

**Keywords:** Children, Autism spectrum disorders, Anxiety symptoms, Video game, Cognitive-behavioral therapy

## Abstract

The aim of the present study was to examine the additive effect of elements of cognitive behavioral therapy (CBT) on the video game Mindlight in decreasing anxiety of children with an autism spectrum disorder (ASD). A non-concurrent multiple baseline design with 8 children with ASD in the age of 8–12 was used. CBT did not have the hypothesized additive effect on Mindlight in decreasing anxiety of children with ASD. Instead, multiple participants already experienced a decrease in anxiety during the Mindlight sessions. Yet, several participants did experience a stabilization in anxiety at a low level during the CBT sessions. For now, it can be concluded that CBT does not have an additive effect on Mindlight.

Video games have the potential to enhance mental health and well-being in children and adolescents (Granic et al. 2014; Ferguson and Olson 2013). The applied video game Mindlight was developed for treatment of anxiety symptoms and disorders in children. Recent studies have shown that Mindlight was equally effective as a control game, as well as to the Dutch translation of the CBT group treatment protocol Coping Cat (Nauta and Scholing 1998) in decreasing anxiety symptoms over time (Schoneveld et al. 2016; Schoneveld et al. 2018). A more recent randomized controlled trial (RCT; Wijnhoven et al. 2020) tested whether Mindlight was effective in decreasing anxiety symptoms in children with autism spectrum disorder (ASD) in a clinical setting. This study showed that Mindlight was more effective than a control game in decreasing parent-rated anxiety symptoms. Yet, the intervention was not more effective than a control game in decreasing child-rated anxiety symptoms. Based on experiences during the RCT, it was hypothesized that adding elements of cognitive behavioral therapy (CBT) could further enhance the effect of Mindlight. Therefore, the aim of the present—non-concurrent multiple baseline—study was to examine the additive effect of CBT elements on Mindlight in decreasing anxiety symptoms of children with ASD in a clinical setting.

Mindlight aims to tackle anxiety in children by using exposure to threatening cues (Abramowitz et al. 2011; Craske et al. 2014), neurofeedback to help train children to regulate arousal levels associated with anxiety (Hammond 2005) and attention bias modification (Muris and Field 2008; see study protocol for more information about Mindlight: Wijnhoven et al. 2020). There are several reasons why Mindlight could be effective in decreasing anxiety in children with ASD. It is known that children with ASD profit more from visual prompts and structured sensory information than from verbal information (Johnco and Storch 2015; Silver and Oaks 2001). Mindlight is a computer based intervention that uses visual aids and structured sensory information to train emotion regulation skills (e.g., relaxation). Moreover, in the treatment of children with ASD it is important to translate their special interests in metaphors (Johnco and Storch 2015). Therapists could use the metaphors in Mindlight (e.g., the main character ‘Arty’) to explain therapeutic content, to reinforce treatment participation and to build a therapeutic relationship. Finally, children with ASD have difficulties with recognizing and expressing their thoughts and feelings (White et al. 2009). Mindlight is an experiential game, which means that it makes children aware of their physical and emotional feelings and the way in which they could alter these feelings.

Despite the effective and suitable treatment elements in Mindlight, it is still unclear how to design therapy sessions in clinical practice in order to maximize the effect of the game intervention on anxiety symptoms of children with ASD. Results of the RCT (Wijnhoven et al. 2020) showed that child-rated anxiety symptoms decreased during the game sessions, but increased again between post-intervention and the 3-months follow-up. This is in line with outcomes in the study of McNally Keehn et al. (2013), who also found an overall decrease in child- and parent-rated anxiety symptoms in children with ASD after a CBT program (‘Coping Cat’), but a specific increase in anxiety between post-intervention and 2-months follow-up. One of the reasons for this, is that the nature of anxiety symptoms and, in turn, the treatment needs of children with ASD can vary to a great extent (Kerns et al. 2016). Another explanation for this increase might be that children with ASD do not automatically know how to use the coping skills they learned during the game in scary situations they encounter in daily life. Research indeed showed that children with ASD have difficulties with generalizing skills they learned in therapy to multiple contexts in daily life (White et al. 2009; McNally Keehn et al. 2013). Craske et al. (2014) suggested that anxiety regulation skills need to be practiced in multiple fearful contexts in order to generalize and remain effective. This indicated that children who played Mindlight need to practice the skills they learned in multiple daily life situations (e.g., at school and during social activities) in order to generalize the learned coping skills and to maintain long-lasting effects.

However, little is known about how to establish optimal generalization of the coping skills children learned in the video game to daily life situations. Swan et al. (2016) stated that coping skills are adaptive ways of processing and reacting to internal and external stressors that could be learned in therapy, but that these skills would also need to be applied and practiced in daily life. Moreover, the authors argued that the generalization of coping skills to daily life could be maximized by the therapist by providing elements of cognitive behavioral therapy (CBT) that focus on altering cognitive biases and dysfunctional behavior. These elements could be used to increase the reinforcement of desired behavior, stimulating practice in multiple anxiety-provoking situations and to increase the use of reminders of learned skills (e.g., ‘coping thoughts’; Swan et al. 2016).

These insights could also be used to maximize the generalization of the learned coping skills in Mindlight to daily life. It is argued that CBT elements could be used by the therapist to explore together with the child in what way they could practice coping skills they learned in daily life (e.g., at school) by using the experiences during the gameplay. Fernando et al. (2015) showed that using a combination of video game sessions and therapist-guided CBT improved therapy effects and enhanced treatment adherence in adolescents with bulimia nervosa when compared with treatment as usual. It was expected that the addition of CBT elements to the Mindlight sessions would maximize therapeutic effects on anxiety symptoms of children with ASD.

## Theoretical Background Mindlight-CBT Sessions

Cognitive biases form an important underlying mechanism of anxiety symptoms in children (Waters et al. 2008). These biases comprise an increased attention to threatening cues and a fearful interpretation of these cues, which elicits feelings of anxiety in children. Research has shown that these biases are also present in anxious children with ASD (Luxford et al. 2017). Therefore, it is important to target both the attention bias and interpretation bias in treatment of anxiety symptoms in children with ASD.

In the game Mindlight, children are exposed to threatening cues (e.g., monsters) and they experience how they can regulate their anxiety during exposure by neurofeedback (Hammond 2005). Moreover, attention bias modification puzzles are aimed at letting children experience how to specifically search for positive stimuli in an environment with both positive and negative or threatening stimuli, which has shown to be important in anxiety treatment (Muris and Field 2008; Waters et al. 2008, 2018). Altogether, Mindlight is an experiential way of learning how to cope with anxious thoughts and feelings, in which children do not actively need to reflect on this learning process. However, reflection on this learning process could result in generating effective ‘coping thoughts’ that might improve the generalization of the coping skills that children with ASD learned in Mindlight to daily life (Swan et al. 2016).

In the CBT-sessions, reflection is introduced by the therapist by exploring the interpretation biases of the child during the gameplay. This can be realized by helping the child in expressing the experienced anxious cognitions and feelings during the play of Mindlight. Also, therapist and child could discuss in which way the child decreased or altered these anxious cognitions during the game, to help modify the interpretation biases of the child. By using the experiences during Mindlight, this reflection process could be made easier, more vivid and more engaging for the child than in ‘normal’ CBT-sessions, which has shown to be important in children with ASD (Johnco and Storch 2015). In turn, they could examine how they could use the skills they learnt in the game in daily scary situations to alter the anxious cognitions (cognitive restructuring; Waters et al. 2008) into the earlier described ‘coping thoughts’ (e.g., ‘When I breath calmly in and out, I can do this’). In their homework (exposure) exercises, children could practice the skills they learnt in scary situations at school, at home and at social activities, which in turn could improve their overall coping skills in multiple scary situations in daily life (Craske et al. 2014; Swan et al. 2016). Moreover, parents are involved in the CBT-sessions to stimulate parents to support their children in practicing learned coping skills at home (Swan et al. 2016), which has shown to be especially important in CBT for children with ASD (Storch et al. 2013). By adding CBT elements to the Mindlight sessions, attention biases and interpretation biases are targeted: Mindlight targets the attention bias of children towards negative or threatening cues by attention bias modification puzzles and CBT targets the interpretation bias of children by training children how to use ‘coping thoughts’ when facing a threatening or negative cue. Eventually, this could lead to a better generalization of coping skills to daily life and to a higher total decrease of anxiety symptoms in children with ASD.

## Design and Hypotheses

The aim of this study was to examine the potential additive effect of evidence-based CBT elements (cognitive restructuring and exposure to multiple daily situations) on Mindlight in decreasing child-rated anxiety symptoms of children with ASD and normal cognitive functioning in a clinical setting. Because the effect of Mindlight has already been tested in previous work (Wijnhoven et al. 2020), the present study focused specifically on the possible additive effect of CBT in a preliminary way, and by taking an individual rather than a group-approach. Moreover, it was tested whether perceived coping skills of children showed a higher increase during CBT compared to Mindlight. To study this, a non-concurrent multiple baseline design was used (see Onghena and Edgington 2005; Smith 2012). Because daily assessments were administered, the course of the anxiety symptoms could be investigated in a more elaborate way. Moreover, coping skills were assessed, resulting in a more extensive analysis of the potential working mechanisms of Mindlight and CBT. It was expected that CBT elements would increase the effect of Mindlight on child-rated anxiety symptoms of children with ASD. Moreover, it was expected that perceived coping skills of children showed a higher increase during CBT compared to Mindlight exclusively.

## Methods

### Procedure

**Fig. 1:**
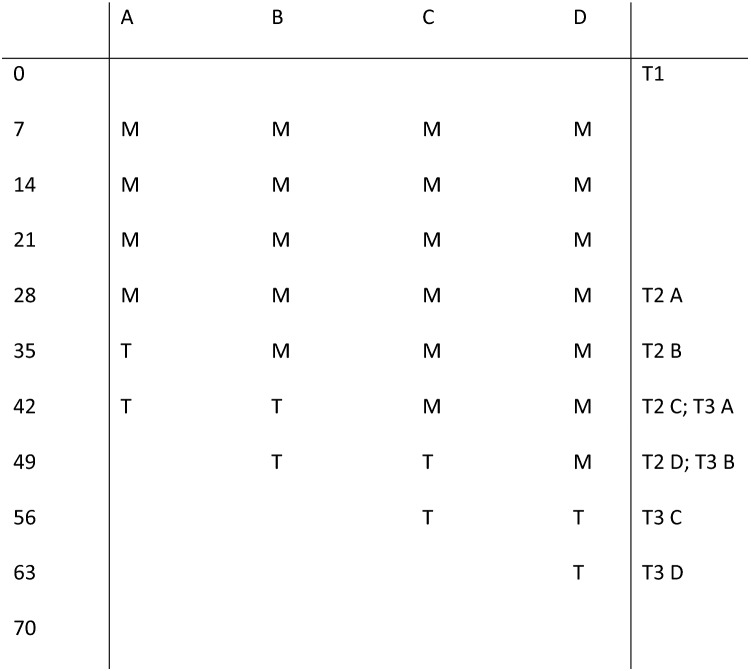
Overview of daily measurements (0–70) and the time points for the start of Mindlight (M), CBT (T) and T1–T3 in all four baselines (A = 4, B = 5, C = 6, D = 7 Mindlight sessions)

A medical ethics committee approved the current study (NL50023.091.14) and all procedures were in accordance with the 1964 Helsinki declaration. In the concurrent multiple baseline design that was used (Onghena and Edgington 2005; Smith 2012; see Fig. 1), participating children were randomly assigned to four different lengths (A–D) of baseline periods (Baseline phase; M). These baseline periods consisted of playing Mindlight in weekly sessions of 1 h. After the Mindlight sessions (Baseline phase; M; 4–7 sessions), participants received two weekly CBT sessions of 1 h (Treatment phase; T; 2 sessions). By offering Mindlight in the baseline phase and CBT sessions in the treatment phase, the additive effect of CBT elements on Mindlight could be investigated. By randomly determining the start of the CBT sessions, it was possible to statistically control for external factors such as therapeutic attention, repeated testing and maturation.

In the present study, N = 8 children with ASD in the age of 8–12 years old participated. This age range was chosen, because Mindlight was specifically designed for decreasing anxiety symptoms in children in this specific age range. Context of recruitment was a mental health institute (GGZ Oost Brabant) in the Netherlands. To determine eligibility, parents and children filled in a screening (T0) on anxiety symptoms (SCAS-C for children; Scholing et al. 1999a; SCAS-P for parents; Scholing et al. 1999b). If children had at least subclinical anxiety symptoms, they were approached for participation. When children and parents agreed to participate, active written informed consent of the parents was obtained. Participating children were randomly assigned to a baseline period of 4–7 weekly Mindlight sessions (see Fig. 1: A = 4, B = 5, C = 6, D = 7 Mindlight sessions). In total, two children were randomly assigned to each baseline length. Children and parents rated the child’s anxiety level on a scale of 0–10 on a daily basis during 10 weeks (70 days) after the first baseline assessment, which took place 1 week before the start of Mindlight. Primary and secondary outcomes were assessed before the start of Mindlight (T1; see Fig. 1), after the last Mindlight session (T2), after the last CBT session (T3) and at 3-months follow-up (T4). Moreover, parents underwent a semi-structured interview (ADIS-P; Siebelink and Treffers 2001) at T1 and at T4 to determine the remission rates of the anxiety disorders that are described in the Diagnostic and Statistical Manual of Mental Disorders 4th Edition-Text Revision (DSM-IV-TR; American Psychiatric Association 2000).

### Participants

In total, 8 children (7 boys and 1 girl) of 8–12 years old with a diagnosis of an ASD participated in the present study. ASD diagnoses were based on psychological and/or psychiatric assessment of the DSM-IV (American Psychiatric Association 2000) criteria for Autistic Disorder, Asperger’s Disorder or PDD-NOS. This assessment was carried out by a clinical expert who conducted a diagnostic assessment that was adapted to the diagnostic ‘needs’ of the individual child and for example consisted of a developmental anamnesis with parents and/or standardized observation of the child with the Autism Diagnostic Observation Scale (ADOS; Bildt et al. 2013). The other inclusion criterion was the presence of at least subclinical anxiety symptoms, defined by mean + 1 SD on the total score and/or one or more subscales (OCD subscale excluded) of the SCAS-C and/or SCAS-P (Muris et al. 2000; Nauta et al. 2004). Exclusion criteria were absence of parental permission and presence of prominent suicidal ideation or other severe psychiatric problems that need immediate treatment (e.g., severe trauma). Moreover, children with ASD who already received treatment for their anxiety symptoms were excluded. Receiving treatment for other ASD-related symptoms was not an exclusion criterion. All participants (and/or their parents) received psychological treatment for other ASD-related symptoms, except for emotion regulation difficulties because of the possible treatment overlap with elements in Mindlight or CBT. In total, five children had an additional diagnosis of attention deficit hyperactivity disorder and one child had an additional diagnosis of specific phobia and social anxiety disorder. Four out of eight children received pharmaceutical treatment for their ASD- and/or ADHD-related symptoms. All participating children were in primary school, with two children following special education. The total IQ of all children was > 85. All children were of Dutch origin. Table 1 shows the profile of subclinical and clinical anxiety symptoms of the participating children, based on the scores on the SCAS-C/P at T0 and the ADIS-P at T1.

**Table 1:** Participants’ profile of subclinical and clinical anxiety symptoms based on the scores on the SCAS-C/P at T0 and the ADIS-P at T1

	Separation anxiety	Social phobia	Specific phobia	Generalized anxiety	Panic disorder/agoraphobia
Participant 1					
Subclinical				X	
Clinical		X	X		
Participant 2					
Subclinical	X			X	X
Clinical			X		
Participant 3					
Subclinical	X				
Clinical			X	X	
Participant 4					
Subclinical					
Clinical		X	X	X	
Participant 5					
Subclinical	X			X	
Clinical		X	X		
Participant 6					
Subclinical					
Clinical		X			
Participant 7					
Subclinical					X
Clinical		X	X	X	
Participant 8					
Subclinical					X
Clinical		X	X		

### Primary Outcome Measure

#### Child-Rated Anxiety Symptoms

The Dutch translation of the Spence Children’s Anxiety Scale (SCAS; Scholing et al. 1999a) was used to measure child-rated anxiety symptoms. The SCAS consists of 44 items (e.g., ‘I am afraid when I have to sleep alone’, ‘I worry about things’) on a 4-point scale, ranging from ‘never’ to ‘always’. Scores on items ranged from 0 to 3, with higher scores indicating more anxiety symptoms. The scale consists of six subscales that correspond with the different anxiety disorders that are described in the DSM-IV: panic/agora phobia, separation anxiety, social phobia, generalized anxiety, obsessive compulsive anxiety and anxiety for physical injury. The SCAS has good validity and reliability (Muris et al. 2000; Spence et al. 2003). The SCAS has good validity and reliability (Muris et al. 2000; Spence et al. 2003). Moreover, the predictive validity of the SCAS-C/P in the ASD population was moderate-good and the internal consistency was excellent (Carruthers et al. 2020). The mean Cronbach’s Alpha over T0–T4 was .88.

Moreover, anxiety symptoms were assessed with daily questions that children had to answer via the e-mental health platform of the mental health agency where they were recruited. At the end of every day, they had to rate their anxiety (‘How anxious/nervous did you feel today?’) and their happiness (‘How happy did you feel today?’) during that day on a scale from 0 to 10, with a higher score indicating respectively more anxiety or happiness. Moreover, the daily questionnaire contained five filler items about the time spent on daily activities of the child (e.g. ‘How many hours did you play with other children today?’).

### Secondary Outcome Measures

#### Parent-Rated Anxiety Symptoms^1^

The Dutch translation of the Spence Child Anxiety Scale for Parents (SCAS-P; Scholing et al. 1999b) was used to measure parent-rated anxiety symptoms. The SCAS-P consists of 38 items on a 4-point scale ranging from 0 (never) to 3 (always). The items and subscales of the SCAS-P correspond with the items of the child version of the SCAS. Only items referring to an internal state (e.g., item 4: ‘I feel afraid’) were rephrased into observable behaviour for parents (e.g., ‘My child complains of feeling afraid’). The SCAS-P consists of the same six subscales as the child version. The SCAS-P has good reliability and validity (Nauta et al. 2004). The mean Cronbach’s Alpha over T0–T4 was .70.

Furthermore, parents also had to answer daily questions on the anxiety symptoms of their child via the e-mental health platform of the mental health agency where they were recruited. At the end of every day, they had to rate the anxiety (‘How anxious/nervous did your child feel today?’) and happiness (‘How happy did your child feel today?’) of their child during that day on a scale from 0 to 10, with a higher score indicating respectively more anxiety or happiness.

#### Anxiety Disorders

The Dutch translation of the Anxiety Disorders Interview Schedule for DSM-IV, Parent version (ADIS-P; Siebelink and Treffers 2001) was used to assess the presence of anxiety disorders in the participating children. This is a semi-structured diagnostic interview focusing on parents that can be used to diagnose anxiety disorders in children of 7–17 years old. The interview consists of standardized questions, with ‘yes’, ‘no’ and ‘different’ as possible answers. On basis of the answers, the interviewer has to give his/her clinical judgement and decision about the presence and severity of every disorder. In this study, the presence of the following DSM-IV anxiety disorders was assessed: separation anxiety disorder, social phobia, specific phobia, panic disorder, agoraphobia and generalised anxiety disorder. The interview was administered by a qualified therapist or by a master student under supervision of a qualified therapist. The ADIS-P has good psychometric properties (Siebelink and Treffers 2001). Moreover, the study of Lecavalier et al. (2014) showed that the ADIS-P was an appropriate measure for the ASD population.

##### Coping Skills

The Dutch translation (CSLK; De Boo and Wicherts 2007) of the Coping Strategies Checklist for Children (CCSC-R1; Ayers and Sandler 1999) was used to assess the self-reported coping skills of the participating children. The CSLK consisted of 54 items (e.g. ‘When I have problems or difficulties…..I listen to music; I do not think about it’) on a 4-point scale, ranging from ‘hardly ever’ to ‘almost always’. The CSLK consisted of five subscales with a further division of several subscales: Problem focused coping, Positive cognitive reframing, Distraction strategies, Avoidance Strategies and Support Seeking Strategies. Scores on items ranged from 1 to 4, with higher scores indicating that the corresponding coping strategy is more present in a child. The CSLK has shown to be a valid and reliable questionnaire (De Boo and Wicherts 2007). There are no psychometric properties of the CSLK for children with ASD. The mean Cronbach’s Alpha over T1–T4 was .91.

### Treatment Protocol Mindlight-CBT Sessions

Because the intervention took place in a mental health agency, it was conducted by qualified psychologists, or by master students who were supervised by qualified psychologists. First, children played Mindlight (M; see Fig. 1) for 1 h per week during the baseline period at the recruitment location. In the Mindlight sessions, the therapist gave an introduction (e.g., instructions) and conclusion (e.g. discussion learning points). During the game, the therapist stayed in the same room as the children, but could only be approached for questions or help. This session protocol was identical to the protocol that was used in the RCT (Wijnhoven et al. 2015). After the last Mindlight session, children received two CBT-sessions (T). In the first CBT-session, the therapist and child discussed anxious thoughts and feelings that were experienced during the game. Moreover, they discussed how the child reduced his/her anxious thoughts and feelings during the game. After that, the therapist guided the child in making the translation from the strategies that were used by the child to reduce anxiety during the game into ways in which the child could use these skills (e.g., exposure, cognitive restructuring and relaxation) in anxious situations in daily life. Finally, the therapist and child together created a homework exercise in which the child should practice the skills that were learned in the game in one or more anxious situations in daily life (e.g. at school or at home). In the second CBT-session, the therapist and child discussed how the homework exercise went and what the child learned from the exercise. Moreover, they examined how the child could continue with practicing the learned skills in anxious situations in daily life after the end of the CBT-sessions. Parents were invited to join the two CBT-sessions during the last 15 min in order to stimulate and help the child with creating homework exercises and to think about ways in which they could support their child in executing the homework exercises. The content, amount and duration of both the Mindlight and CBT sessions was feasible for all participating children.

### Sample Size

In a study aimed on power in single case series designs (e.g., multiple baseline design), in which the power of designs with different numbers of participants (3–7) and assessments were compared, it was found that a number of data points (assessments) of 40 and higher resulted in sufficient statistical power, regardless of the number of included participants (Heyvaert et al. 2017). In the current study, eight participants were included and data were collected in 70 daily assessments per participant, resulting in sufficient statistical power.

### Statistical Analysis

Randomization tests (Bulté and Onghena 2008) were conducted with the SCRT package (Edgington and Onghena 2007) that was integrated in a web-app (‘Shiny app’; De et al. 2017) to analyze the difference in decrease of the daily measured anxiety symptoms between the baseline phase (Mindlight; M; see Fig. 1) and treatment phase (CBT; T) over all eight participants. Because randomization tests do not rely on a random sampling assumption, they can provide a better and more reliable alternative than parametric statistical tests for analyzing data from single-case (series) designs.

**Fig. 2:**
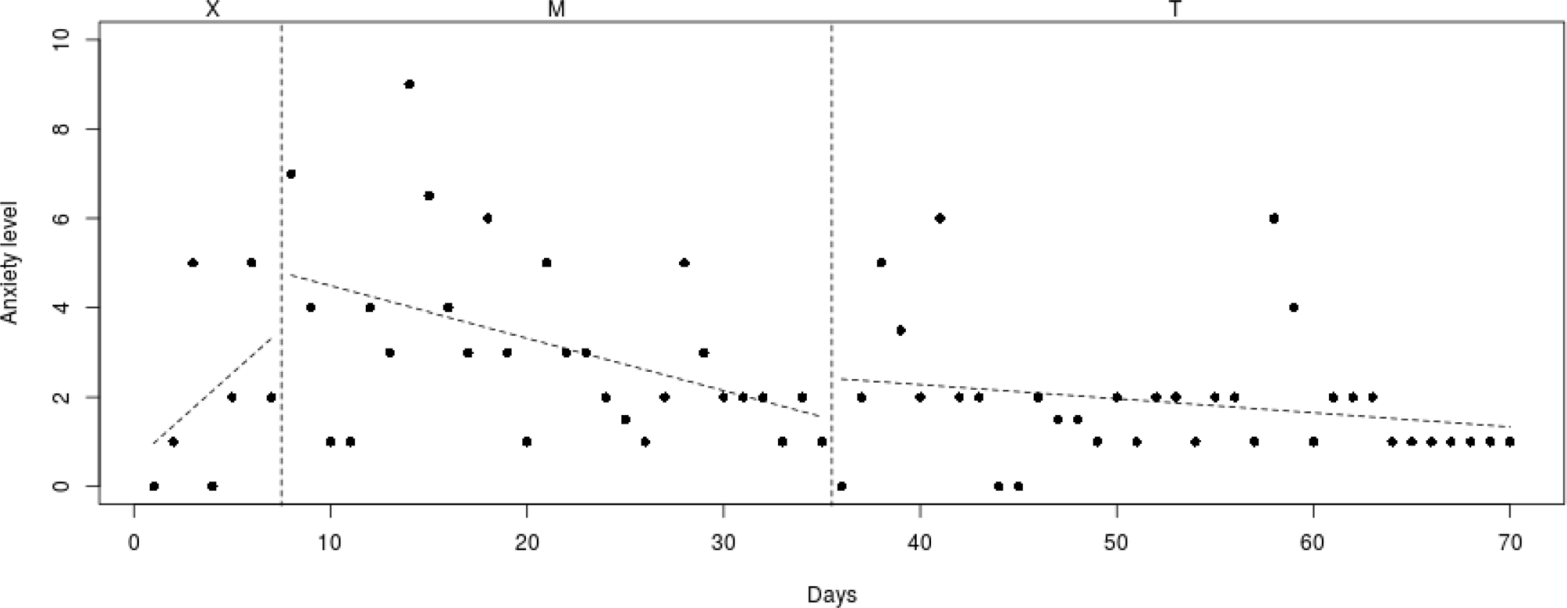
Course of daily measured anxiety level (0–10) over 70 days for participant 1

**Fig. 3:**
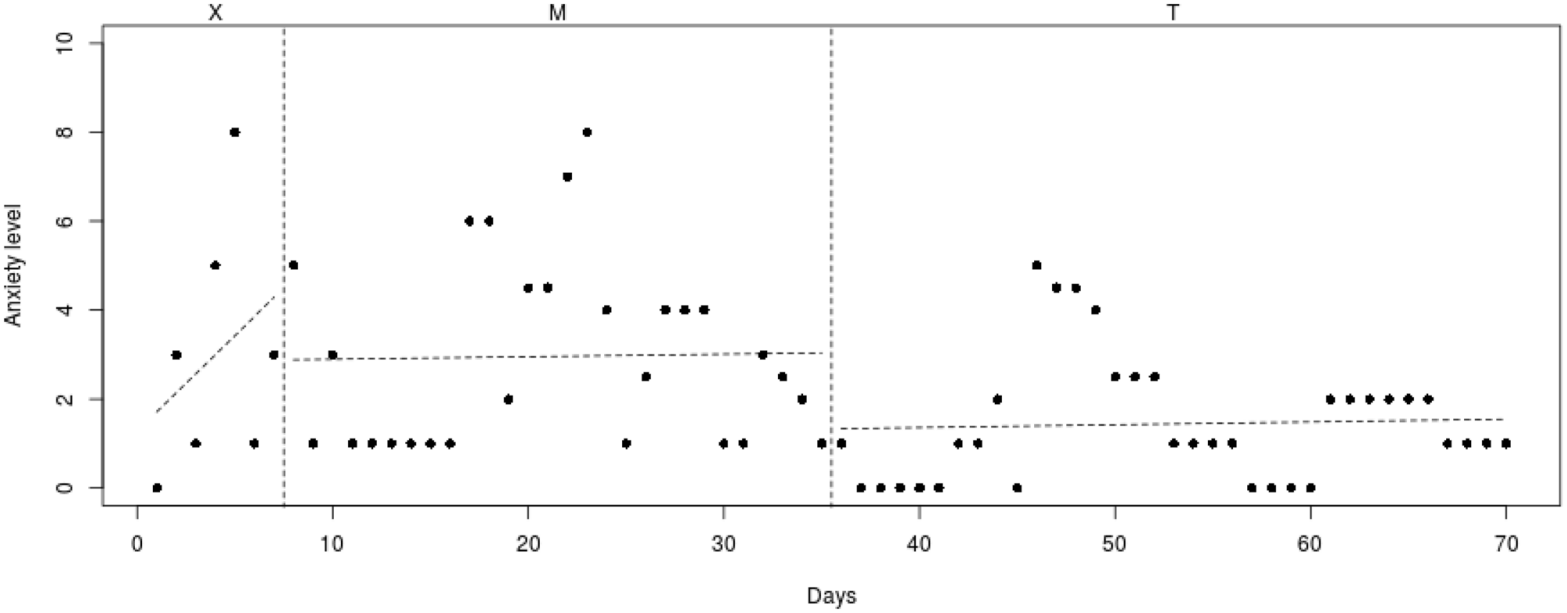
Course of daily measured anxiety level (0–10) over 70 days for participant 2

**Fig. 4:**
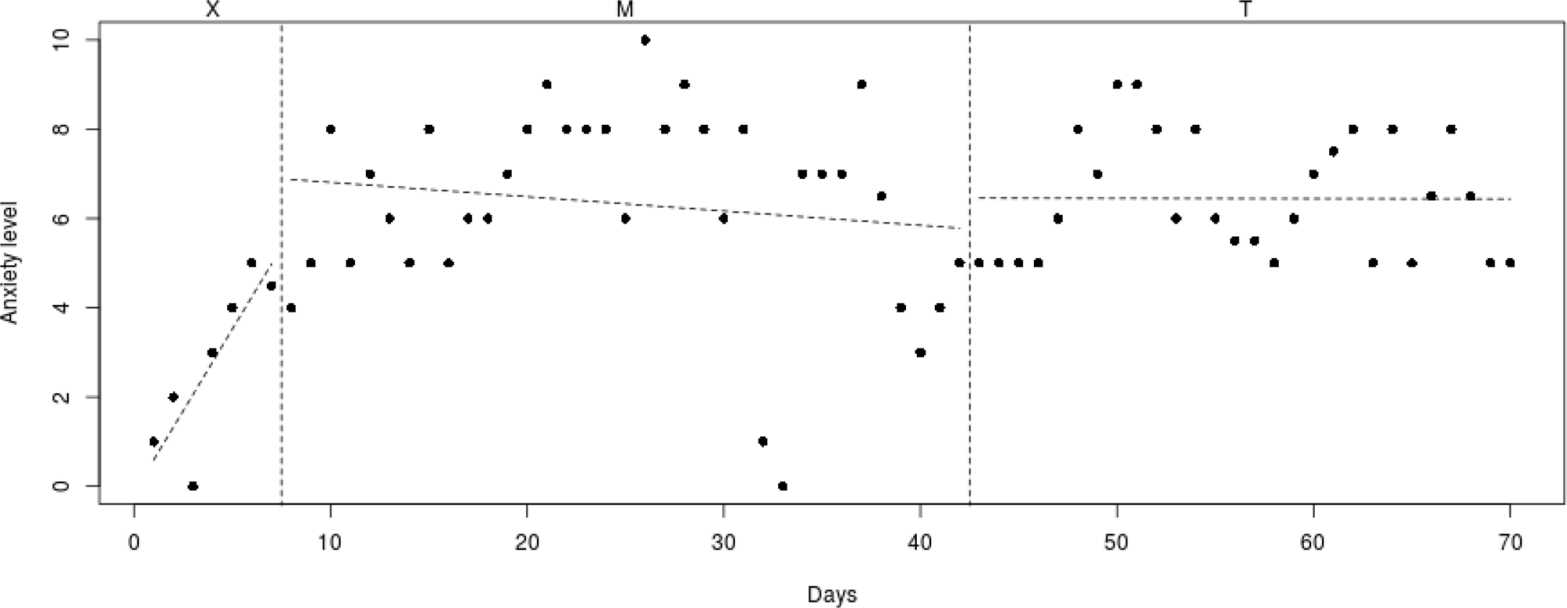
Course of daily measured anxiety level (0–10) over 70 days for participant 3

**Fig. 5:**
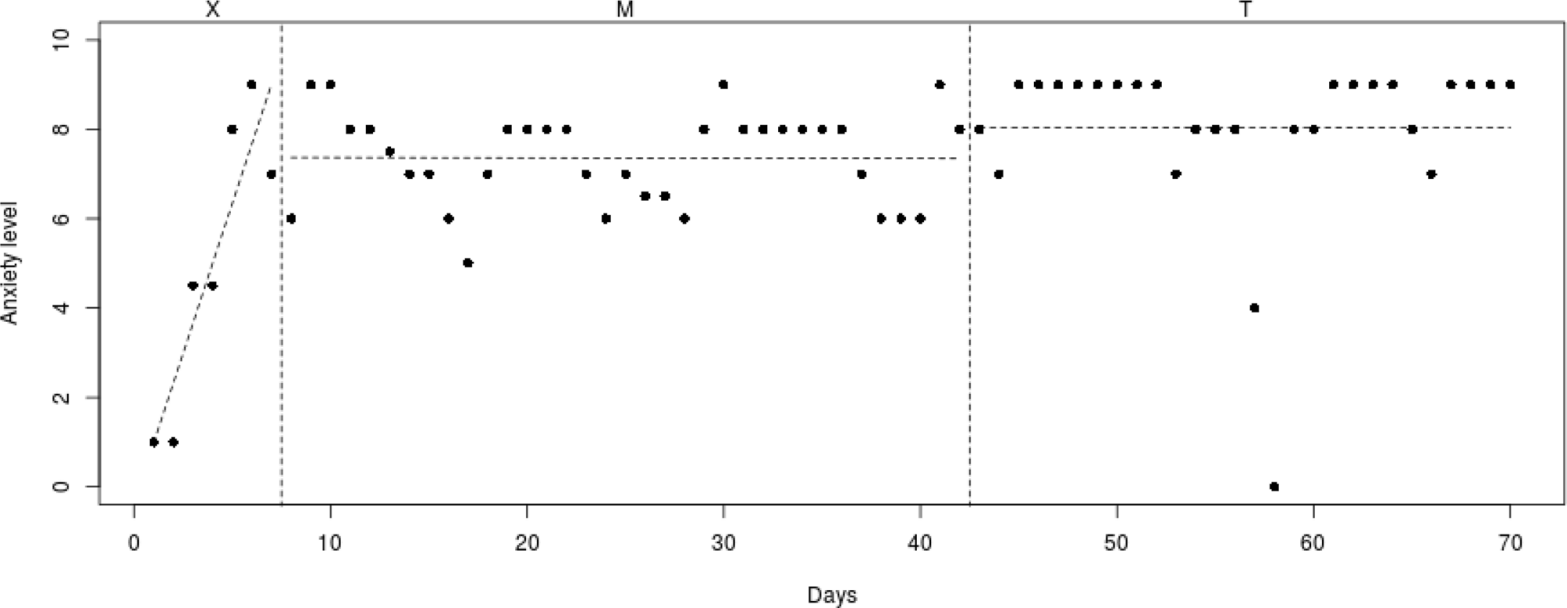
Course of daily measured anxiety level (0–10) over 70 days for participant 4

**Fig. 6:**
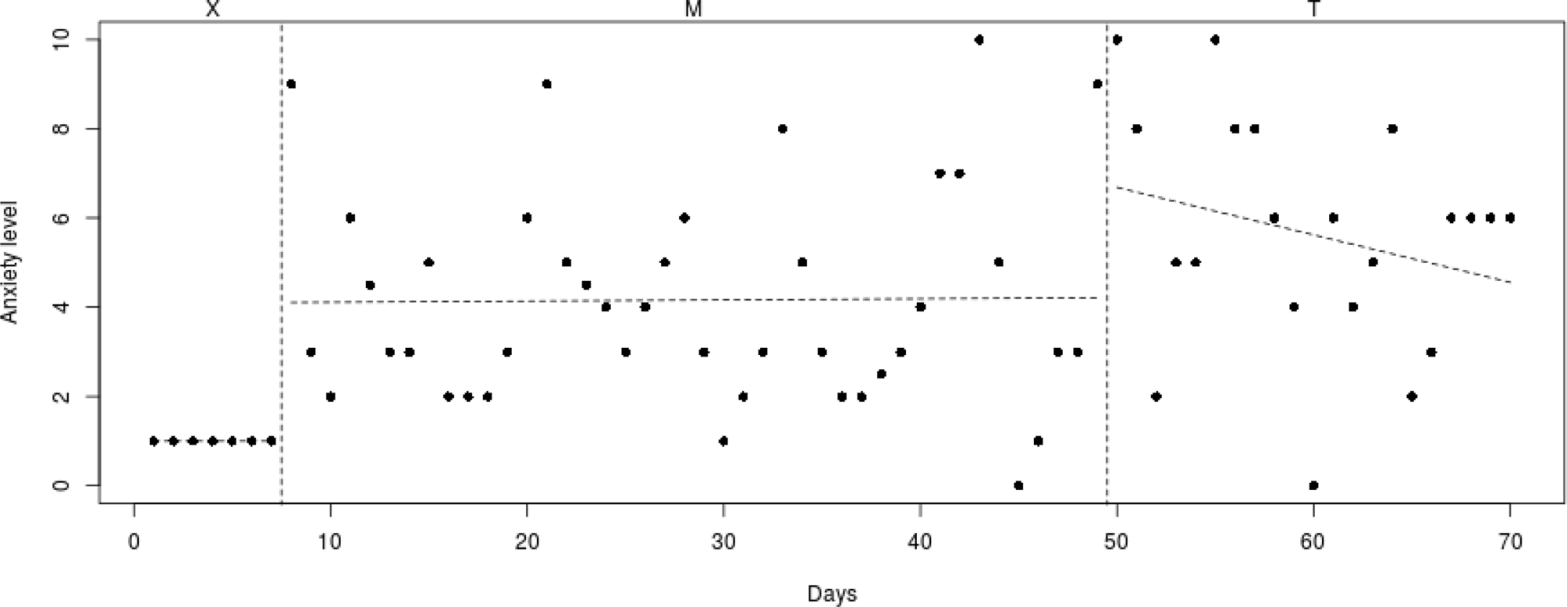
Course of daily measured anxiety level (0–10) over 70 days for participant 5

**Fig. 7:**
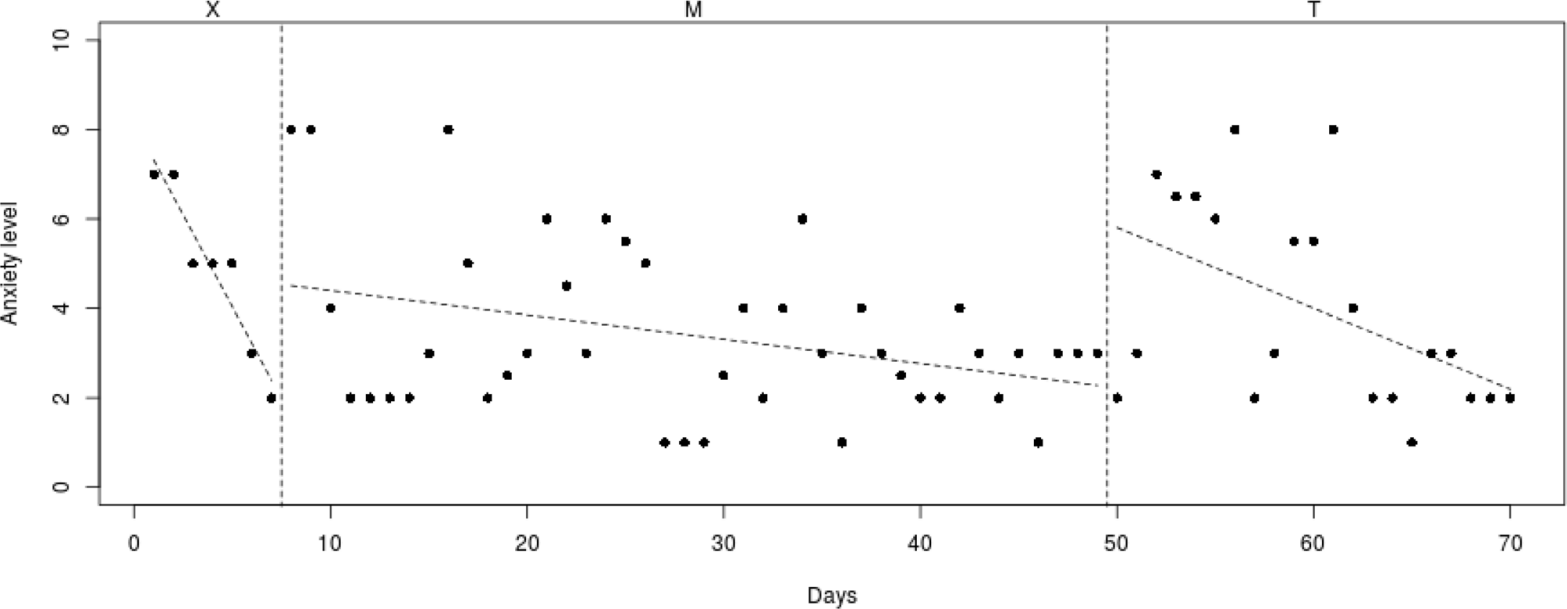
Course of daily measured anxiety level (0–10) over 70 days for participant 6

**Fig. 8:**
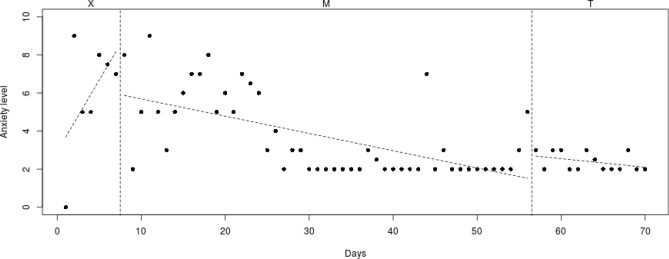
Course of daily measured anxiety level (0–10) over 70 days for participant 7

**Fig. 9:**
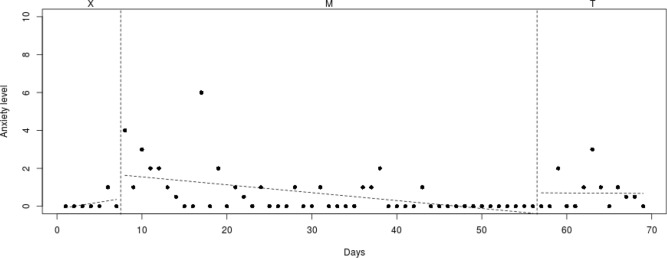
Course of daily measured anxiety level (0–10) over 70 days for participant 8

To analyze individual differences in decrease of anxiety symptoms, the course of the daily measured anxiety symptoms (see Figs. 2, 3, 4, 5, 6, 7, 8, 9) was visually analyzed for every participant, with phase X (7 days before start of Mindlight), phase M (Mindlight/Baseline phase) and phase T (CBT/Treatment phase). The dots indicate the exact anxiety level on a particular day (0–10), the dotted lines indicate least squares regression trends over the different phases (X, M and T). So, visual analyses could show whether anxiety symptoms decreased in a meaningful way in the different phases and could therefore provide insight into the extent to which change could be attributed to Mindlight and CBT (Lane and Gast 2014). Moreover, to investigate whether anxiety symptoms measured by the SCAS-C significantly decreased over time (T0–T4), the Reliable Change Index (RCI; Jacobson and Truax 1991) was calculated for each participant. The RCI was calculated by dividing the difference in total scores on the SCAS-C (T0–T4) by the standard deviation (SD) of the total scale of the SCAS-C as reported in the study of Muris et al. (2000). An RCI > 1.96 on a level of α = .05 (two-sided test) indicated a significant change over time (Jacobson and Truax 1991). Moreover, the RCI’s were calculated for the differences in SCAS-C scores between T0 (screening) and T4 (3-months follow-up), between T1 (pre-test) and T2 (post-Mindlight), between T2 and T3 (post-CBT) and between T3 and T4 (3-months follow-up), to investigate for each participant whether the change in anxiety symptoms and coping skills took place in specific phases of the study. Finally, the remission rates of the anxiety disorders in the ADIS-P (Siebelink and Treffers 2001) were described for each participant.

## Results

### Statistical Outcomes

The Randomization tests were conducted to investigate whether the decrease of the daily measured anxiety symptoms in the CBT phase (T) was significantly different from the decrease of the daily measured anxiety symptoms in the Mindlight phase (M) over all eight participants. Results of the Randomization tests showed that there was no significant difference between the decrease in anxiety symptoms in the CBT phase (T) and the Mindlight phase (M). In other words, results showed that CBT elements had no significant additive effect on decreasing anxiety symptoms next to Mindlight (*p* > .05).

### Summary of Clinical Outcomes

Table 2 shows the differences and RCI’s in SCAS scores (total scale) over the course of the study (screening through 3-months follow-up), between pre-test and post-Mindlight, between post-Mindlight and post-CBT and between post-CBT and 3-months follow-up for every participant. Results showed that participant 1 and 2 experienced a significant decrease in anxiety symptoms over the total course of the study. Moreover, participant 5 showed a significant decrease in anxiety symptoms between the end of Mindlight and the end of CBT. All other participants did not show any significant decrease.

**Table 2:** Differences and RCI’s for SCAS scores between T0 and T4 (screening-3 months FU), T1–T2 (pre-test–post-Mindlight), T2–3 (post-Mindlight–post-CBT) and T3–4 (post-CBT—3 months FU) for every participant

	pp1		pp2		pp3		pp4		pp5		pp6		pp7		pp8	
	Diff	RCI	Diff	RCI	Diff	RCI	Diff	RCI	Diff	RCI	Diff	RCI	Diff	RCI	Diff	RCI
T0–T4	12	2.15	20	3.58	0	.00	4	.72	2	.36	5	.90	7	1.25	9	1.61
T1–T2	5	.90	1	.18	4	.72	3	.54	− 4	− .73	− 2	− .36	0	.00	7	1.25
T2–T3	− 3	− .54	6	1.08	8	1.43	3	.54	14	2.54	1	.18	4	.72	3	.54
T3–T4	5	.90	6	1.08	− 6	− 1.08	6	1.08	− 4	− .73	− 4	− .72	− 3	− .54	0	.00

*Diff* difference

RCI > 1.96 indicates a clinically significant decrease

According to the ADIS-P that was administered at pre-test and at 3-months follow-up, participant 1, 3, 7 and 8 remitted from several or all specific phobias, participant 3 remitted from generalized anxiety disorder and participants 6 and 8 remitted from social phobia at 3-months follow-up.

Finally, the results of the CSLK showed that participant 1, 2, 7 and 8 showed a significant increase in coping skills between pretest and 3-months follow-up or between post-Mindlight and post-CBT. For participant 2 and 8, positive coping skills significantly increased until the 3-months follow-up. Participant 1 and 7 showed a significant decrease in positive coping skills from the end of the CBT-sessions to 3-months follow-up. Moreover, participant 6 showed a significant decrease in the coping skills direct problem solving and analyzing the problem over time. Finally, the participants (3, 4 and 5) who did not experience a decrease in anxiety over time, showed only small improvements in their coping skills.

### Individual Outcomes

#### Participant 1

The course of daily measured anxiety symptoms of participant 1 is shown in Fig. 2. Visual analysis showed that daily measured anxiety symptoms increased in the week before Mindlight (phase X) and that the anxiety symptoms showed a clinically relevant decrease (from mean score 5 to mean score 2) during the Mindlight sessions (phase M). The anxiety symptoms of participant 1 slightly decreased during the CBT sessions (phase T) and showed a stabilization at a low level from week 50. The results of the SCAS-C showed a significant decrease in anxiety symptoms from screening through 3-months follow-up (RCI: 2.15 > 1.96; see Table 1). The ADIS-P that was administered with the parents of participant 1 showed the same diagnoses at 3-months follow-up as at pretest (social phobia and specific phobia), but the number of specific phobias decreased from three (doctors/dentist, birds, dressed up people) to only one (doctors/dentist).

#### Participant 2

The course of daily measured anxiety symptoms of participant 2 is shown in Fig. 3. Visual analysis showed that daily measured anxiety symptoms increased in the week before Mindlight (phase X) and that the anxiety symptoms did not decrease during the Mindlight sessions (phase M). During the CBT-sessions (phase T), the anxiety symptoms did not decrease but showed a lower and more stable level than during the Mindlight sessions (from mean score of 3 to mean score of 1.5). The results of the SCAS-C showed a significant decrease in anxiety symptoms from screening through 3-months follow-up (RCI: 3.58 > 1.96; see Table 1). The ADIS-P that was administered with the parents of participant 2 showed no difference in diagnoses between pretest and 3-months follow-up.

#### Participant 3

The course of daily measured anxiety symptoms of participant 3 is shown in Fig. 4. Visual analysis showed that daily measured anxiety symptoms increased in the week before Mindlight (phase X) and continued to be at a high level (mean score of 6/6.5) during the Mindlight (phase M) and CBT sessions (phase T). The results of the SCAS-C showed that there was no significant decrease of anxiety symptoms from screening through 3-months follow-up (see Table 1). In contrast, results of the ADIS-P showed that participant 3 met the criteria of generalized anxiety disorder and specific phobia at pretest, but did not meet the criteria of any anxiety disorders at 3-months follow-up.

#### Participant 4

The course of daily measured anxiety symptoms of participant 4 is shown in Fig. 5. Visual analysis showed that daily measured anxiety symptoms increased in the week before Mindlight (phase X) and continued to be at a high level (mean score of 7/8) during the Mindlight (phase M) and CBT sessions (phase T). The results of the SCAS-C showed that there was no significant decrease of anxiety symptoms from screening through 3-months follow-up (see Table 1). Results of the ADIS-P showed that there was no difference in diagnoses of anxiety disorders between pretest and 3-months follow-up.

#### Participant 5

The course of daily measured anxiety symptoms of participant 5 is shown in Fig. 6. Visual analysis showed that daily measured anxiety symptoms increased from the week before Mindlight (phase X) through the end of the Mindlight sessions (phase M) and that the anxiety symptoms showed a slight decrease after the start of the CBT sessions (phase T), but did not end at a satisfying low level at the end of the CBT sessions (mean score of 4.5). The results of the SCAS-C showed no significant decrease in anxiety symptoms from screening through 3-months follow-up, but a significant decrease in anxiety symptoms between the end of Mindlight and the end of CBT, which is in line with the course of the daily measured anxiety symptoms (RCI: 2.54 > 1.96; see Table 1). The ADIS-P that was administered with the parents of participant 5 showed no difference in diagnoses between pretest and 3-months follow-up.

#### Participant 6

The course of daily measured anxiety symptoms of participant 6 is shown in Fig. 7. Visual analysis showed that daily measured anxiety symptoms were high at the start of all phases (X, M and T) and decreased during all phases. Anxiety levels were low at the end of the CBT sessions (mean score of 2), but were not stabilized. The results of the SCAS-C showed no significant decrease in anxiety symptoms from screening through 3-months follow-up (see Table 1). Results of the ADIS-P showed that participant 6 met the criteria of social phobia at pretest, but not at 3-months follow-up.

#### Participant 7

The course of daily measured anxiety symptoms of participant 7 is shown in Fig. 8. Visual analysis showed that daily measured anxiety symptoms increased during the week before Mindlight (phase X) and that anxiety symptoms decreased from the start of Mindlight through the end of the CBT sessions (from mean score of 6 to mean score of 2). During the CBT sessions, anxiety symptoms stabilized at a low level. The results of the SCAS-C showed no significant decrease in anxiety symptoms from screening through 3-months follow-up (see Table 1). The ADIS-P that was administered showed that participant 6 met the criteria of social phobia, specific phobia and generalized anxiety disorder at pretest, but did only meet the criteria of social phobia at 3-months follow-up.

#### Participant 8

The course of daily measured anxiety symptoms of participant 8 is shown in Fig. 9. Visual analysis showed that daily measured anxiety symptoms were at a stable low level during all phases (X, M and T). During the Mindlight sessions, anxiety symptoms slightly decreased from a mean score of 2 to a mean score of 0. The results of the SCAS-C showed no significant decrease in anxiety symptoms from screening through 3-months follow-up (see Table 1). The ADIS-P that was administered showed that participant 8 met the criteria of social phobia and specific phobia at pretest, but did not meet the criteria of any anxiety disorder at 3-months follow-up.

## Discussion

The aim of this study was to examine the potential additive effect of CBT on Mindlight in decreasing child-rated anxiety symptoms of children with ASD and normal cognitive functioning in a clinical setting. The additional CBT elements did not have the hypothesized additive effect on Mindlight in decreasing anxiety of children with ASD, which was illustrated by the randomization tests and visual analyses. Moreover, analysis of the SCAS scores at T2–T3 showed that participant 5 was the only participant to show a clinically significant decrease in anxiety symptoms during the CBT sessions.

When examining the daily measured anxiety symptoms in Figs. 2, 3, 4, 5, 6, 7, 8, 9 more in detail, it can be seen that five participants (participant 1, 2, 6, 7 and 8) already showed a decrease in anxiety symptoms during the Mindlight sessions, which is in line with Schoneveld et al. 2016, 2018) and Wijnhoven et al. (2020). Moreover, three of these participants (1, 2 and 7) showed a pattern of stabilization of anxiety symptoms at a low level during and after the CBT-sessions. Analysis of the SCAS scores indicated that two of these participants (1 and 2) experienced a clinically significant decrease in anxiety symptoms over the course of the study, and that four of these participants (1, 6, 7 and 8) remitted from one of more anxiety disorders at 3-months follow-up. This indicates that five participants seem to have benefited from the Mindlight sessions and that the CBT-sessions had a stabilizing effect on anxiety symptoms in three of these five participants.

However, for three participants (3, 4 and 5) visual analysis showed that neither Mindlight nor CBT had a decreasing effect on their anxiety symptoms. This finding was partly supported by both the analysis of the SCAS scores and the remission rates at 3-months follow-up. Participant 5 did show a clinically significant decrease in anxiety symptoms during the CBT-sessions, but the anxiety level at the end of the CBT-sessions was still high, indicating that this decrease was not clinically satisfying. In addition, participant 3 remitted from generalized anxiety disorder and specific phobia at 3-months follow-up, while visual analysis of the daily measured anxiety symptoms did not show improvements in his anxiety level and the RCI of the SCAS scores at T3–T4 even showed an increase in anxiety symptoms. This could be explained by the difference in outcomes of anxiety assessment with a questionnaire (SCAS-C) and an interview (ADIS-P), which has been shown to lead to different anxiety ratings (Van Steensel et al. 2011).

Moreover, it was expected that perceived coping skills of children showed a higher increase during CBT compared to Mindlight. On basis of the results, it can be concluded that it is likely that in four participants the CBT-sessions have contributed to an increase in coping skills and in turn to a stabilization in anxiety symptoms at a low level, and that in two of these participants the learned coping skills were also generalized to daily life situations (in line with Craske et al. 2014; Swan et al. 2016).

The difference in outcomes between participants could be explained by several possible factors. When comparing the SCAS scores at T0 and T1, it can be seen that those children who showed a decrease in anxiety already showed a decrease in anxiety symptoms (except for participant 8) between screening and pretest, and that the children that did not show a decrease in anxiety symptoms showed an increase in anxiety symptoms between screening and pretest. It is possible that the waiting time between screening and pretest had an anticipation effect on the anxiety symptoms of the improvers (Ahola et al. 2017), which means that anticipation of treatment may have prepared the participants for the intervention by activating therapeutic processes such as increasing awareness of their anxiety symptoms (Arrindell 2001), installation of hope (Dowling and Rickwood 2015) and increasing expectations of treatment effect (Thiruchselvam et al. 2019). Furthermore, it is remarkable that participant 1 and 2 showed the highest overall improvements on anxiety symptoms, anxiety disorders and coping skills, because these children received the smallest amount of Mindlight sessions (4 sessions). This is in line with the study of Stice et al. (2009), showing that a shorter program duration was associated with a better treatment outcome. Especially for children with ASD this might be true, considering the effort and energy that it costs for these children to engage in a large number of therapy sessions because of their social and cognitive difficulties (Johnco and Storch 2015). For these children, it might be more important to invest in practicing coping skills in multiple daily life situations (as suggested by Craske et al. 2014) than to follow a long treatment protocol. Finally, the overall lack of a stable decrease in anxiety symptoms over time might be due to the presence of multiple psychiatric diagnoses (e.g., ASD and ADHD) in most of the participating children.

This study has a few limitations. First, because some children already showed a decrease in anxiety symptoms during the Mindlight sessions (baseline), it is statistically more difficult to find a significant additive effect of CBT on Mindlight compared to studies in which the baseline period did consist of a waiting time (e.g. Spuij et al. 2013). However, the results of the visual analyses and the clinical outcomes provided a more in-depth and balanced view of the results by showing the individual trajectories. Second, despite the use and comparison of multiple methods, the visual analysis of data is a qualitative analysis method and in this way rather subjective.

Overall, it could be concluded that CBT did not have a significant additive effect on Mindlight in decreasing anxiety symptoms of children with ASD. Instead, multiple participants already experienced a decrease in anxiety symptoms during the Mindlight sessions, which is in line with the decreasing anxiety symptoms in previous studies on Mindlight (Schoneveld et al. 2016, 2018; Wijnhoven et al. 2020). Yet, several participants did experience a stabilization in anxiety symptoms at a low level during the CBT sessions in combination with an increase in coping skills. These children might have practiced the skills they learned during Mindlight and CBT in multiple situations in daily life, which may have led to an improvement of their overall coping skills and in turn to a decrease in anxiety symptoms (Craske et al. 2014; Swan et al. 2016). Children who did not show a decrease in anxiety symptoms may not have been able to improve their coping skills, for example because of a lack of practice in daily life situations. Alternatively, the combination of Mindlight and CBT may not be fulfilling treatment expectations and needs for these children, leading to a lack of decrease in anxiety symptoms.

The study has some clinical implications. It showed that for some children with ASD, treatment consisting of only Mindlight might be sufficient to decrease their anxiety symptoms, while for only few children the addition of CBT may be useful. There are also children with ASD that do not benefit from Mindlight and CBT at all and need other types of treatment. This confirms the well-known fact that the population of children with ASD is heterogeneous, both in its clinical presentations and its treatment needs. It requires sufficient clinical expertise to be able to obtain that information that is necessary to obtain the optimal adjustment to the child’s needs and ultimately a personalized treatment. Future research should provide better insight into the individual factors that could predict which type of children with ASD benefit from which kind of treatment. For now, it can be concluded that CBT does not have an additive effect on Mindlight, but might be a useful addition to Mindlight for at least some children with ASD.

## Figures and Tables

**Fig. 10:**
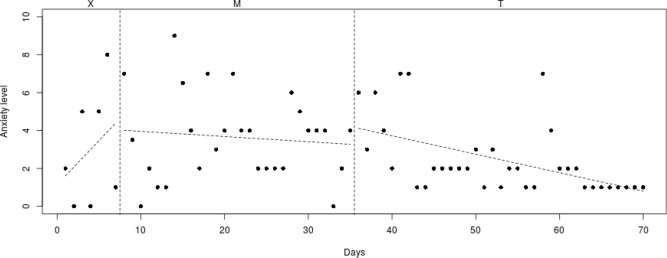
Course of parent-rated anxiety level (0–10) over 70 days for participant 1

**Fig. 11:**
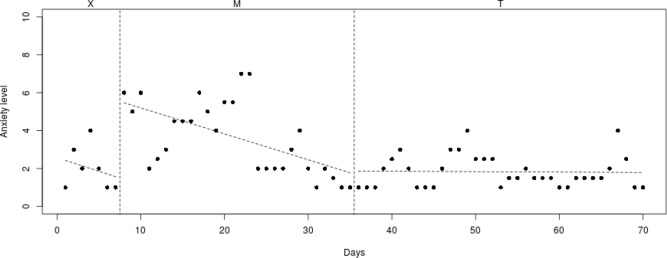
Course of parent-rated anxiety level (0–10) over 70 days for participant 2

**Fig. 12:**
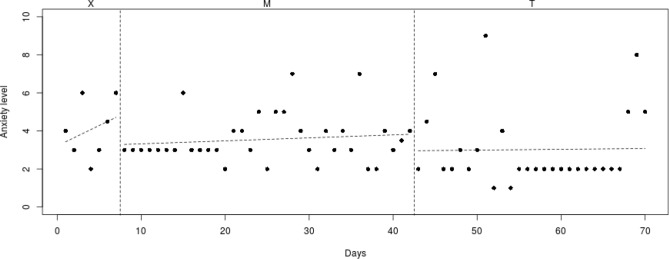
Course of parent-rated anxiety level (0–10) over 70 days for participant 3

**Fig. 13:**
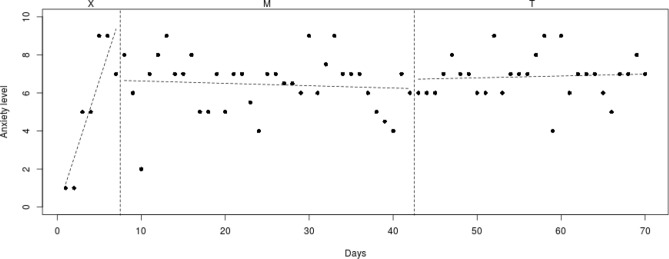
Course of parent-rated anxiety level (0–10) over 70 days for participant 4

**Fig. 14:**
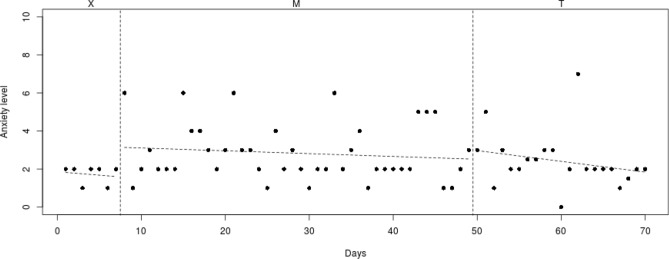
Course of parent-rated anxiety level (0–10) over 70 days for participant 5

**Fig. 15:**
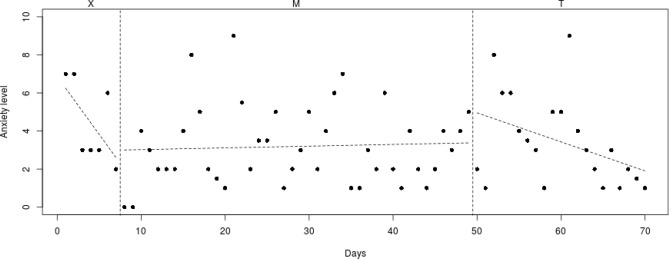
Course of parent-rated anxiety level (0–10) over 70 days for participant 6

**Fig. 16:**
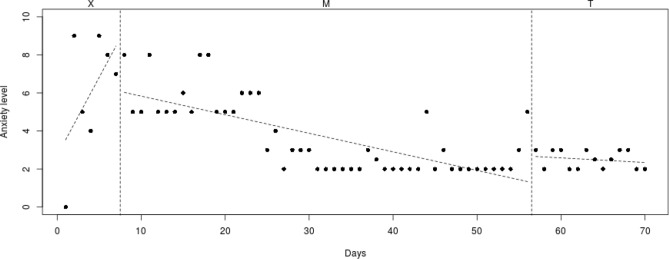
Course of parent-rated anxiety level (0–10) over 70 days for participant 7

**Fig. 17:**
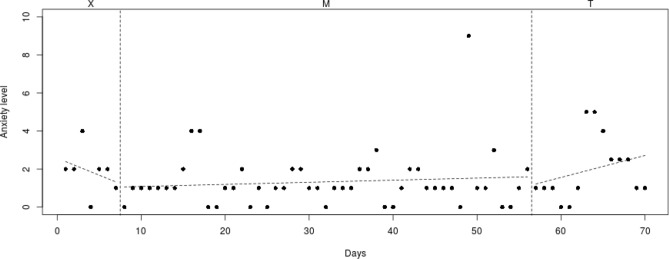
Course of parent-rated anxiety level (0–10) over 70 days for participant 8

## Appendix

In this Appendix, graphs (Figs. ^10^, ^11^, ^12^, ^13^, ^14^, ^15^, ^16^, ^17^) of the parent-rated daily measured anxiety level of the participating children are presented and discussed. For most part, results of the parent-rated daily measured anxiety level resemble the results of the child-rated daily measured anxiety level (Figs. 2, 3, 4, 5, 6, 7, 8, 9). Results of Randomization tests showed that there was no significant difference between the decrease in parent-rated anxiety symptoms in the CBT phase (T) and the Mindlight phase (M). In line with the results of the child-rated anxiety symptoms, CBT had no significant additive effect on parent-rated anxiety symptoms next to Mindlight (*p* > .05).

When examining the individual outcomes, it can be seen that the course of the parent-rated anxiety level over 70 days largely resembles the course of the child-rated anxiety level. Like in the child-rated results, participant 1, 2, 6 and 7 showed a decrease in anxiety symptoms over time. This decrease was more supported by the RCI’s of the parent-rated SCAS scores (see Table

**Table 3:** Differences and RCI’s for parent-rated SCAS scores between T0 and T4 (screening-3 months FU), T1–T2 (pre-test–post-Mindlight), T2–3 (post-Mindlight–post-CBT) and T3–4 (post-CBT—3 months FU) for every participant

	pp1		pp2		pp3		pp4		pp5		pp6		pp7		pp8	
	Diff	RCI	Diff	RCI	Diff	RCI	Diff	RCI	Diff	RCI	Diff	RCI	Diff	RCI	Diff	RCI
T0–T4	14	2.57	8	2.05	− 3	− .55	8	1.47	3	.71	14	2.57	19	3.49	7	1.29
T1–T2	4	.74	− 5	− 1.18	7	1.29	3	.55	− 4	− .95	− 6	− 1.10	9	1.65	− 1	− .18
T2–T3	6	1.10	9	2.13	2	.37	1	.18	− 2	− .47	9	1.65	0	.00	1	.18
T3–T4	− 2	− .37	− 15	− 3.55	− 7	− 1.29	6	1.10	5	1.18	− 1	− .18	0	.00	7	1.29

*Diff* difference

RCI > 1.96 indicates a clinically significant decrease

3) than the RCI’s of the child-rated SCAS scores (see Table 2). As can be seen in Table 3, results for participant 1, 2, 6 and 7 showed a clinically significant decrease of parent-rated anxiety symptoms (measured with SCAS) between screening and 3-months follow-up, while for child-rated anxiety symptoms this was only the case for participant 1 and 2. Only participant 8 showed no decrease in anxiety over time, but in the child-rated results it can be seen that the decrease for participant 8 was also small due to the low starting level of anxiety.

For participant 1 and 2, a difference was found in the timing of the decrease of anxiety symptoms. For the parent-rated anxiety symptoms of participant 1, a higher decrease was found in the CBT phase (T), while for the child-rated anxiety symptoms a higher decrease was found in the Mindlight phase (M). For participant 2, the Mindlight phase showed a higher decrease of parent-rated anxiety symptoms compared to the child-rated anxiety symptoms. Surprisingly, RCI’s of the parent rated SCAS scores showed a clinically significant decrease in anxiety symptoms between T2 (post-Mindlight) and T3 (post-CBT) and not between T1 (pre-test) and T2 (post-Mindlight), which would have been in line with the high decrease of parent-rated daily measured anxiety symptoms. However, for participant 2 the daily measured anxiety symptoms were continued to be measured during three weeks after T3 (post-CBT assessment), which showed that the high decrease during the Mindlight sessions and the first week of the CBT-sessions did not persist after the last CBT session, but showed a slightly unstable pattern. The RCI’s of participant 2 also showed a negative RCI between T3 (post-CBT) and T4 (3-months follow-up), indicating an increase in anxiety symptoms. Possibly, the slightly unstable pattern after the last CBT session was the beginning of an increase in anxiety symptoms till 3-months follow-up. Finally, for participant 3 and 5 parents rated the level of anxiety symptoms lower than children over the whole course of the study.

## Footnotes

1 Parent-rated results were presented in the “Appendix” section.
